# The Book Dog and Semiotic Resources in Envisionment Building of a Text World

**DOI:** 10.1007/s10936-018-9617-0

**Published:** 2018-12-03

**Authors:** Ewa Bergh Nestlog, Helene Ehriander

**Affiliations:** 1 Department of Swedish, Linnæus University, 351 95 Växjö, Sweden; 2 Department of Film and Literature, Linnæus University, 351 95 Växjö, Sweden

**Keywords:** Literacy, Reading dog, Book dog, Children’s literature, Children’s reading, Meaning-making, Text movability, Envisionment building, Dyslexia

## Abstract

The Linnaeus University project “The Book Dog and Astrid Lindgren” seeks to bring children and literature together and to use the dog as a tool for this. The method involves children reading aloud to trained dogs, called *book dogs*. By studying the practice of the book dog, we seek more profound knowledge of the importance of the reading practice for children’s reading. Such knowledge can have didactic implications for reading practices also in contexts where there is no book dog. In the study reception theories (Langer in Envisioning knowledge. Building literacy in the academic disciplines, New York, Teachers College Press, 2011a; Langer in Envisioning literature. Literary understanding and literature instruction, 2nd ed., New York, Teachers College Press, 2011b) are developed with perspectives of discourse analysis (Fairclough in Discourse and social change, Polity Press, Cambridge, 1992). and social semiotics (Halliday in Language as social semiotics. The social interpretation of language and meaning, Edward Arnold, London, 1978). The result shows that the dog contributes with semiotic resources in the meaning-making process; the text world comes to life for the child through the expanded envisionment building where the dog is central. Since pupils read texts in all school subjects, the study should be relevant for all types of teachers when shaping reading practices that support pupil’s meaning-making, also in contexts where there is no book dog. The study can also say something about what engagement, attentiveness, and non-judgemental attitudes can mean for pupils, even they in reading and writing difficulties (Bergh Nestlog and Ehriander 2016).

When a reader gets really involved in a story and perhaps even becomes totally absorbed in a book, it is a sign of a successful meeting between reader and text, where the reader has entered the text world and moves around in it. In this article we present a pilot study where we investigate how Ronja, 8 years old, creates meaning in and from a text that she reads aloud together with a book dog and its handler.

Programmes to promote literacy by letting a child read aloud to a specially trained dog and its handler have become increasingly popular in many countries, above all in the USA and Britain, through an organization called R.E.A.D. (Reading Education Assistance Dogs). In Sweden, the word *läshund* (reading dog) was included in the list of new words for 2012 by the Swedish Academy, after the media had been alerted to the methods used by this organization. The Linnaeus University project “The Book Dog and Astrid Lindgren”, financed by the State Inheritance Fund, seeks to bring children and literature together and to use the dog as a tool for this. The dogs that have been trained to work in the project are called *book dogs*. The project aims to stimulate the joy of reading in an appealing way and to spread knowledge about children’s literature in general and Astrid Lindgren’s works in particular. It is aimed at children with difficulties in reading, writing, and speech, children with poor motivation to read, and children who already think it is fun to read and who can contemplate reading more, along with the adults who are in contact with these children. The method involves children reading aloud to a dog (Ehriander 2015a, b, 2017). Dogs are undemanding and non-judgemental, and they have a soothing effect on the children who read aloud, an activity that many children can otherwise find stressful (Friesen 2012). As part of the project 25 dogs have had 1 year of training and are now working in schools and libraries in different parts of Sweden.^1^

Dog handlers, teachers, and children testify that reading aloud to a dog gives positive results in motivation for reading, reading development, and self-esteem, but not much has been written about how reading aloud in this situation actually functions and what it is that so often brings positive results.

The aim of this study is therefore to use the experiences of one child’s reading together with a book dog in order to find theoretically based explanations for the child’s expressions of meaning-making in text and practice. Another aim is to understand the significance of the book dog for the child’s reading. By studying the practice of the book dog, we seek more profound knowledge of the meaning of the reading practice for the child’s reading. Such knowledge can have didactic implications for reading practices in contexts where there is no book dog. Since pupils read texts in all school subjects, we think that the study is relevant for all types of teachers when shaping reading practices in their subjects, not primarily so that they will use book dogs, but to understand more about the meaning of supporting pupils in envisionment building while reading. The study can also say something about what engagement, attentiveness, and non-judgemental attitudes—as shown by the book dog and the handler—can mean for pupils and especially for those in reading and writing difficulties (Bergh Nestlog and Ehriander 2016).

To understand Ronja’s meaning-making during reading, we proceed from reception theories (Langer 1995, 2011a, b) which we develop with perspectives of discourse analysis (Fairclough 1992) and social semiotics (Halliday 1978). Linking up with New Literacy Studies, we apply a broad understanding of the concept of reading as participation in social practices (Luke and Freebody 1999, pp. 5–8).

## Previous and Future Research

Lori Friesen, who gained her doctorate in 2012 at the University of Alberta with the dissertation *Grade 2 children experience a classroom*-*based animal*-*assisted literacy mentoring program: An interpretive case study*, studies how children perceived what these reading sessions together with the dog team meant for them, and what the relationship to the dogs was like for children who read in school, and who came into contact with the dogs in connection with special support. Friesen’s conclusion is that:

animal-assisted literacy mentoring programs can offer children valuable forms of social, emotional, and academic support in the classroom context. Specifically, four main themes emerged inductively from the data: (1) Animal-assisted literacy sessions drew the consistent and enthusiastic participation of all of the children in the classroom and were viewed as anticipated escapes from typical school routines; (2) These sessions invited playful, imaginative literacy teaching and learning opportunities for group participants; (3) Novel and familial modes of interrelationship within these sessions transformed the network of relationships among group members, and finally; (4) The students’ positive, transformative associations with literacy in the broader school context and in their home literacy lives collectively contributed to a carnivalesque climate of literacy support. (Friesen 2012, unpaginated abstract)

There have been several studies of how the presence of dogs and interaction with them reduces children’s stress level, for instance measured as the level of cortisol in their saliva (Uvnäs-Moberg 2000). Since children often find that reading, especially reading aloud, is stressful, the presence of the dog helps them to calm down and thus feel more secure in the situation. Working with animals is extremely effective for pupils with concentration difficulties and acting-out behaviour (Katcher and Wilkins 1998; Kaufmann 1997). The dog strengthens the children’s faith in their own ability and hence also their self-esteem. By being with the dogs, children satisfy many physical and mental needs, and by using the dog in school the pupils can be motivated to take part in the teaching even if it feels difficult for them. Pupils who have been in the company of animals often display greater empathy for other people, an important property for working with values in school (Jalongo 2014). Dog handlers in the book dog project testify that the ability to create and maintain social relations can be developed in different ways with the aid of contacts with animals. The increased self-confidence and self-esteem is reflected in the pupil’s school work. Concentration improves, which leads to generally improved study results (Ehriander 2016). Studies show how insecure children and those with emotional difficulties become more relaxed and can withstand stress better with an ordinary dog than with an ordinary human being in a socially stressful situation (Uvnäs-Moberg 2000). In coping with social stress, which reading aloud also entails, the children thus found that the dog was a more effective support than another person was. Mary Renck Jalongo’s article in the journal *Childhood Education* examined the outcome after a 2-year project where pupils have read aloud to a dog (the R.E.A.D. programme). All the participating children considerably improved their reading comprehension and decoding ability. Other indications of a positive development were that the children had less absenteeism in school, used the school library more often, and had better overall grades in school (Jalongo 2005).

In our pilot study the empirical material consists of the experiences gained by a dog handler when a child, Ronja, reads together with the book dog. In focus of the study is the interaction between the data sources—Ronja, the dog and the dog handler. As there are three data sources that form the empirical material for the study, they constitute kind a data triangulation, although they are not studied individually but as a group with the interaction and their joint meaning-making as focal point. Ronja was selected for the study because she is the child in the project who has read to a dog longest, during two long periods over one and a half years. The dog handler has kept notes during the reading sessions. Written informed consent was obtained from the parents of the participant, Ronja, and from herself, for participating in the study. The child’s name has been anonymised.

The project will continue with more study of children reading to the book dog through observations of reading practices and interviews with the children and analysis of texts that the children have written about their reading and about the books. Suitable methods, material, and research questions for coming studies are outlined in Table 1.

**Table 1:** Methods, material, and research questions

Collection method and material	Research questions	Study object: the child, the dog, the dog handler, and children’s texts/drawings
Observations	How does the reader behave in relation to the book text? How does the reader behave in relation to the dog? How does the reader behave in relation to the dog handler? How does the dog handler behave in the reading situation?	Gestures, glances, and other body language Speed and rhythm in speech and movements Speech (and any expressions in writing)
Interviews	How does the reader talk about the text? How does the reader talk about the dog?	The child’s spoken text
The children’s texts	What does the text say about the reader’s meaning-making in the book text and the practice?	The child’s written text

The empirical studies are geared to how the child behaves in speech and writing and how the dog handler uses the dog in the reading situation to support the child in the reading.

## Studying Reading with a Book Dog

Ronja has had two periods of improving her reading together with the trained book dog Arabella. The first period comprised a daily session for 5 weeks and the second period daily for 4 weeks. For roughly 20 min a day Ronja read to the book dog Arabella, and the first book they read together was about how Mimmi’s new neighbour had acquired a dog that he could not look after (*Mimmi och valpen*, (“Mimmi and the Pup”), Gomér 2015). In the last picture Bonita the pup is lying in Mimmi’s bed, and Ronja can heave a sigh of relief—both because the story ended happily and because together with Arabella she has started to overcome her reading difficulties. When the reading has gone on for roughly a month, the dog handler gets a text message from Ronja’s mother, saying that Ronja has suddenly started using her reading ability in everyday life. In the same afternoon she read aloud a recipe on the fridge door and wrote a note to her brother. Ronja no longer tries to avoid anything to do with the written language, having now discovered its potential both as a way to find out something and as a means of communication with others. A few weeks after the first reading period, Ronja says: “I read books now!” and talks about books that she has read in school. They are short, easily read books, but the important thing is that Ronja identifies herself as a reader and a person who finds it fun to read. After a few more weeks Ronja has also become a leisure-time reader, and she can choose to read a book before going to sleep in the evening.

## Theoretical Premises

In this pilot study the primary intention is to find theoretically grounded explanations for one reading child’s expressions of meaning-making in the text and in the practice where the reading takes place.

### Perspectives on Literacy

Literacy in a broad sense is about taking part in activities where one reads and writes, and the ability to create meaning through the written language. To make sense of what one is reading requires both cognitive and social competences. Texts can be viewed as a type of code which has to be decoded in order to create understanding, and if one looks at a text from a social perspective the reader needs to be able to relate to the text and create a personal understanding of what has been read, based partly on previous experience, partly on knowledge about the context to which the text belongs. Then words and sentences can take on a meaning that the reader can understand in terms of its linguistic and social function (Schmidt and Gustavsson 2011, p. 38). With a literacy perspective, reading and writing are regarded as being linked to social practices rather than to people’s individual abilities. With reference to Luke and Freebody (1999, pp. 5–8), literacy includes a repertoire of competences. They write about four resources that interact during reading and other literacy activities: cracking codes,^2^ participating in meaning-making in text, using texts functionally, and critically analysing and transforming texts. With this perspective, reading has to be studied on the basis of the social practice where reading takes place.

### The Social Semiotic Perspective

From a social semiotic perspective we view text as part of a social context where different semiotic resources interact inside and outside the text. Research in social semiotics examines how different modalities such as verbal text, pictures, and sounds can carry information in texts, how the modalities “together create a whole” (Danielsson 2013, p. 169) and how the different modalities function as semiotic resources in learning (Sandström 2015). The concept of semiotic resources concerns “everything we do or use to organize our understanding of the world around us and to communicate” (Danielsson 2013, p. 171). Different media offer possibilities for different semiotic resources. In a hand-written text, sound cannot be available as a resource as it can in a digital text. In this way multimodality is also closely connected to different media. With a social semiotic perspective on learning, the individual is regarded as an active creator of signs, who is in a social context that offers different semiotic resources in meaning-making. When experiences, emotions, thoughts, and knowledge are mediated through different semiotic resources, these become available both for ourselves and for others. By using different semiotic resources, questions and challenges can be represented in such a way as to become alive, rich in perspectives, and palpable. They are thereby also visible and communicable. We communicate and create meaning by using different semiotic resources which are available in the current context.

Kress et al. (2001) focus on the way in which meaning can be expressed through several different modalities such as speech, writing, gestures, visual images, and tactile contact. The different modalities offer different potentials for meaning-making. For example, verbal language is well suited to expressing logical connections, while pictures work well when expressing spatial relations (Danielsson 2013). Many books for children have pictures. Multimodality in texts can increase the reader’s possibilities to interpret and understand the text (Björkvall 2009, p. 6). In the best case the pictures function as a resource for creating meaning in the pupils.

### Dimensions of Discourse and the Reader’s Transactions

**Fig. 1:**
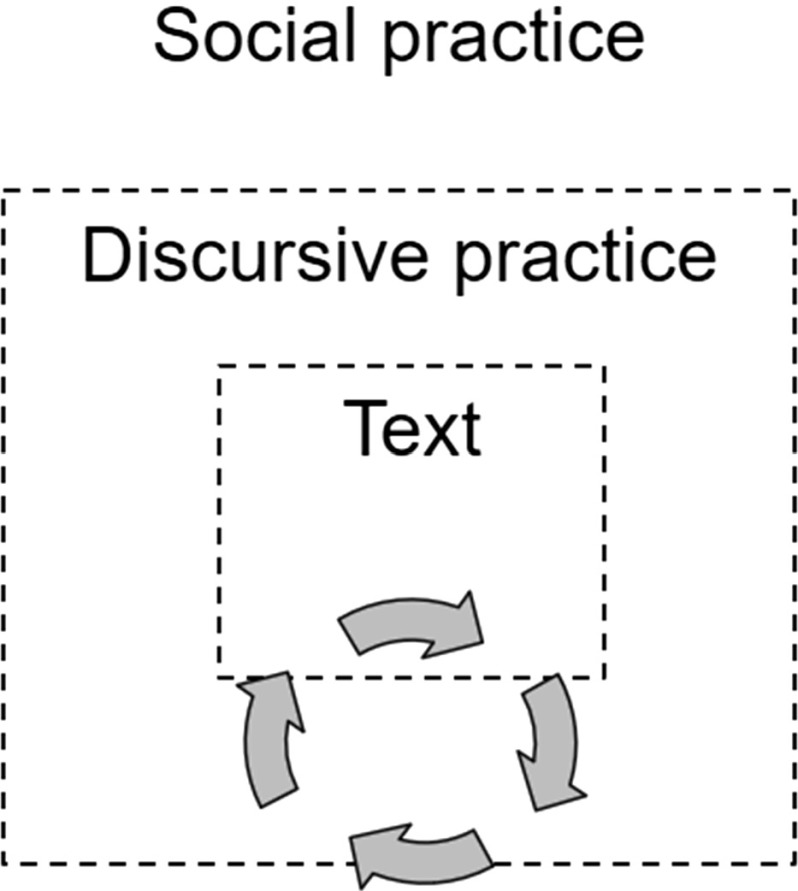
Dimensions of discourse and the reader’s transactions (Bergh Nestlog 2012, p. 48; Fairclough 1992, p. 73)

Social practice is a central concept in theories of critical discourse analysis, where Norman Fairclough’s research occupies a central place (Fairclough 1992, p. 73). The premise there is that discourse means the use of language in speech and writing as a social practice (Fairclough and Wodak 1997). Figure 1 explains how texts are affected by the contexts, that is to say, the practices, in which the texts occur and also that the practices are affected by the texts that are used. Every text (the innermost layer in Fig. 1), oral or written, is part of a discursive practice (the middle layer in Fig. 1), that is to say, a specific and observable context where the text is produced or used. The discursive practice is part of a larger cultural and social context (the outermost layer in Fig. 1), which affects both the discursive practice and the text.

In social practices, conventions develop over time to govern how we behave in interaction with others and how we behave in text, that is to say, how texts and utterances are presented. This means that we express ourselves in different ways, for example, in an academic essay that is to be read by researchers and in a text message sent to a friend. This also explains why there can be a great difference in the language of a private letter written a 100 years ago and one written in our own times. Social practices can also steer the other two dimensions of discourse through laws and ordinances. In school, for example, the curriculum (“social practice” in Fig. 1) can have a direct impact on the teaching (“discursive practice” in Fig. 1) and the texts that are read and written. The focus in this study is not on the social practice, which is outside the discursive practice. What we are concerned with is “text”, namely, the book that Ronja reads, and “discursive practice”, that is, the context where Ronja reads together with the book dog and the dog handler.

One way to understand the reader’s meaning-making together with the book dog is to enlist the aid of the two innermost dimensions of discourse and the transactions—meaning-making processes—performed by the reader in the text and in the discursive practice where the book dog and its handler also participate. The arrows in Fig. 1 represent transactions the reader performed by reading the text and participating in the practice. The transactions are assumed to take place through constant meaning-making as the reader reads, listens, speaks, writes, converses, and acts in connection with the reading. These activities and constant transactions enable rich opportunities for deepening the readers’ meaning-making.

### Movability in Text and Practice—Expanded Envisionment Building

Using theories of reception, researchers study the encounter between text and reader and the reader’s meaning-making through the reading. Judith Langer uses the concept *envisionment building* when she talks of reading comprehension in terms of entering a text world and moving around there (Langer 1995, 2011a, b). Reading is “a changing path in a changing textual landscape” (Liberg 2004, p. 109). Envisionment refers to the reader’s”world of understanding […] at a given point in time” (Langer 2011b, p.10) during or after the time of reading. The original categories used by Langer (1995, 2011a, b) refer to five stances that characterise the envisionment building. We do not use her stances or terms in this study. The concepts in this study are developed under the influence of as well Langer’s reception theory as discourse analytical (Fairclough 1992) and social semiotic (Halliday 1978) theories. In earlier research based on Langer’s theories, the concept of text movability is used as an analytical tool (Bergh Nestlog 2012; Liberg et al. 2011; Hallesson and Visén 2018). When a reader makes a statement about a text and her or his envisionment, one can understand something about the meaning-making that takes place inside the reader. The term *text movability* is used to stress that the reader is the one that is moving, not the text itself. Through three analytical categories—text-based text movability, associative text movability, and transactive text movability—the reader’s statements about the text can be understood and specified. The statements thus display the reader’s text movability. Text-based text movability is closely associated with the text itself; in associative text movability the reader connects envisionment of the text world to experiences from other texts and from his or her own life; and in transactive text movability the reader shows his or her understanding of the text in a broader context which can concern the purpose and function of the text (Bergh Nestlog 2012; Liberg et al. 2011; Folkeryd et al. 2006).

With the concept of text movability as an analytical tool it is possible to investigate Ronja’s reading and reception, that is to say, signs of reading comprehension, of both the envisionment building of the text world of the book and the envisionment building that is open to her in interaction with the dog.

With the help of theories from critical discourse analysis (Fairclough 1992) and social semiotics (Halliday 1978) we develop the reception theories; a start has been made on this in previous studies of meaning-making in pupils in the middle years (Bergh Nestlog 2009, 2012). The movability displayed by the reader in relation to the book is henceforth termed *text movability* and the movability associated with the practice with the book dog is called *practice movability*. By involving the book dog in the concept of text movability we elaborate on the reception theories.

Text movability and practice movability are primarily analysed in terms of statements by the reader and the practice participant, that is to say, Ronja, about the text in the book and about the book dog. We analyse how the reader talks about the text in the book and talks about and with the dog. Besides utterances in verbal language, we also note gestures and gazes, and the speed and rhythm of speech and body expressions that reveal something of Ronja’s movability in the text and in the practice with the book dog (Martinec 2004). When Ronja shows her movability in the expanded envisionment building, she primarily expresses herself orally, but she has also done drawings of herself and the book dog Arabella, and she has written about the thoughts and experiences she had when reading to the dog.

We turn to social semiotic theory and three-dimensional perspectives on language and texts to be able to refine the analyses of the reader’s meaning-making during the reading together with the book dog. This theory assumes that every utterance has three metafunctions (see the three corners in Figs. 3 and 4 below). In an utterance *communication* takes place between the person who says something and the person who reads or listens to the utterance (*interpersonal* metafunction; relations—Who? Why?) about a *content* (*ideational* metafunction; content—What?) put forward through *structures in the text* (*textual* metafunction; linguistic and textual structures—How?) (Halliday 1978).

**Fig. 2:**
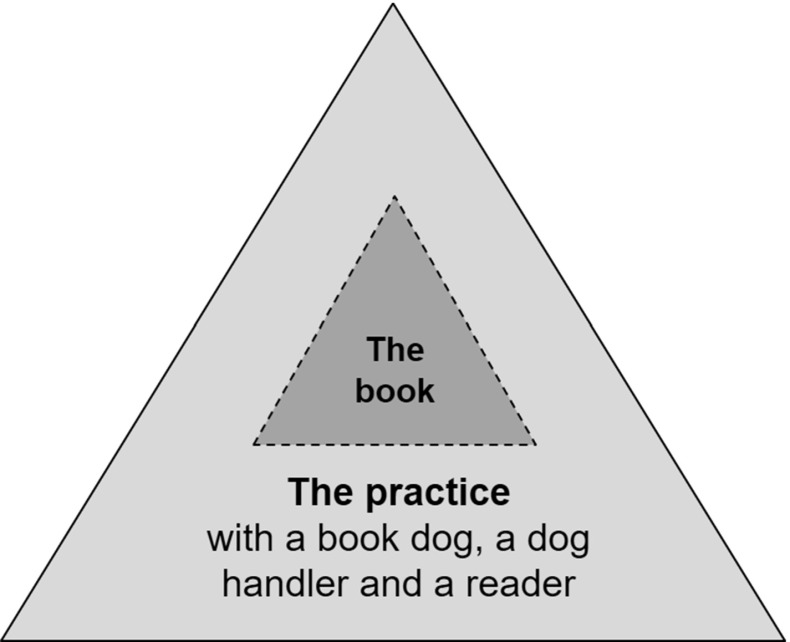
Reading with the book dog—expanded envisionment building

To clarify the relationship between the metafunctions and the discourse dimensions, we have modified the shape of the figure, and instead of square boxes, as in Fig. 1, the discourse dimensions below are drawn as triangles in which each corner represents one of the three metafunctions. In the innermost layer we see the text that is read, in this study the book that Ronja is reading (the dark-grey field in Fig. 2). The innermost layer is the starting point for *the reader’s envisionment building* in which the reader shows text movability. During the reading with the book the dog, the dog handler, and the reader participate in the discursive practice (the light-grey field in Fig. 2).

The participants together constitute the starting point for envisionment building *with the book dog* (the outer layer in Fig. 2). Another way to express oneself about the envisionment building could be to talk about an *expanded envisionment building* in order to underline that they belong together and that the outer gives support to the inner and vice versa.

According to the aim of the study we are trying to find theoretically based explanations for the child’s expressions of meaning-making in text and practice. As shown above, the study draws on some different but compatible theories. The theories are not used separately, instead they are developed to a cohesive system with traces of the original theories, which means that we in the analysis and the discussion use our developed theory as an entity as a whole.

## Ronja’s Text Movability

Ronja reads the text in the book (the innermost discourse dimension in Fig. 1) and creates meaning in the book’s text world. She shows this when she expresses herself about the story and takes an interest in reading more, because she wonders “what happens next”. She also moves in the discursive practice (the discourse dimension in the middle of Fig. 1) during the reading and takes an interest in the dog because “it’s so special to read to Bella”. For Ronja the experience with the book is important and enriching. We see that reading with the dog makes her involved in an expanded envisionment building in which she moves and empathizes, an envisionment that concerns the text in the book and also her companionship with the dog. The latter is created in the discursive practice where the reading takes place. This is factual and concrete, but it is also in some sense an imagination in Ronja that she and the dog handler build up and are part of, together with the dog. We could say that the reader and the dog handler reach an agreement that the dog understands, and they do not question that.

We are constantly making such agreements when we enter literary worlds. For example, we know that Pippi can lift a horse in the stories about Pippi Longstocking. As readers we determine that “this is how it is”, and we reason about and interpret the story on the basis of this agreement. We also note that dog owners, who live in a close relationship with their dog, often chat to the dog and give it a voice. The dog owner *builds an envisionment* where it has been agreed that the dog and the dog owner understand each other, and one can therefore take on the role of the dog’s verbal interpreter.

The dog handler also facilitates communication and contributes to promote Ronja’s text movability by asking questions “for the dog “, for example: “I don’t think Arabella knows that word. Can you help me to explain it to her?” or “Arabella didn’t understand if it was a nice or mean person, can you tell her what you think?” The dog can also borrow the voice from the dog handler, who can talk with the voice of the dog: “I don’t understand why she is angry! Can you please tell me?” In this situation Ronja talks to the dog and not to the dog handler, and explains what the dog is asking for. Ronja and the dog handler can act like this because the common agreement in the silent contract is that the dog listens, speaks and understands. In this way, the dog handler can take away a lot of stress from the situation, which in itself contributes to positive results. Through this method Ronja gets the pleasure to feel smart and helpful as she is a better reader and knows much more than the book dog. In this way the dog handler can discreetly check that Ronja understands the text without her feeling controlled and perhaps failing.

During the reading Ronja shows an explicit interest in the content of the book, and after each page or each spread she comments on the plot and shows clear engagement. She wonders what Mimmi will do, asks why the pup is tied up outdoors, and she speculates about how the story will continue. At times she is engrossed in the book and does not look up when she comments on the action. In this Ronja displays *text*-*based text movability*. In this type of text movability the reader is primarily geared to the *content* of the book by talking about the plot: *What* is the book about? We may thus observe that Ronja has started her envisionment building in the text and displays involvement in the events in the book. With her words and her body Ronja evinces text-based text movability. It is not only through the words in the book that her text movability is expressed. She often uses the illustrations to help in her reasoning about the text and to understand individual words and contexts, as well as to gain support for her own thinking about the text.

Ronja has also made statements about *structures* in the text, that is, *how* the text is written. An example of this type of text-based text movability is seen when Ronja, who finds it hard to distinguish the letters *b* and *d*, read *stängde dörren* (“closed the door”). She explained that she was “trying to make it fit” and that what you close is a *dörr*, not a *börr*. She thus draws the conclusion that it must be *dörr*, even though she is initially uncertain which of the two letters she sees.

**Fig. 3:**
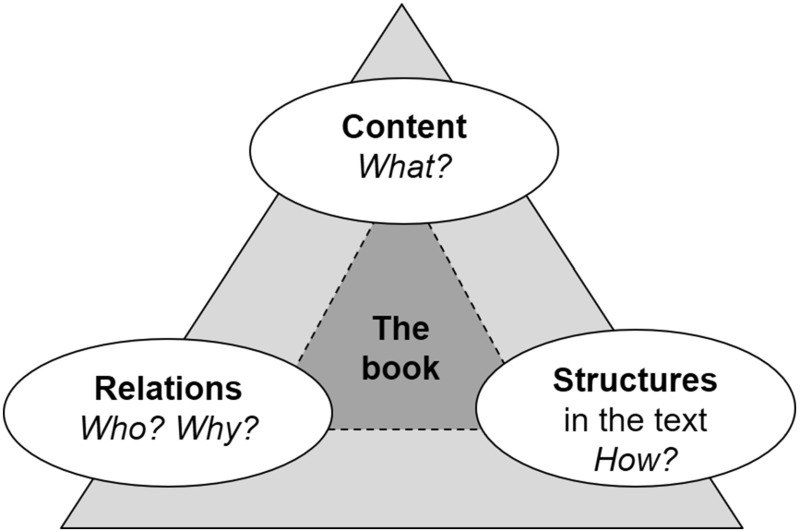
Text movability—envisionment building in the book (cf. Bergh Nestlog 2012, pp. 69 and 138)

Text-based text movability means that the reader is geared to the text itself and to the envisionment building in the text, that is to say, the innermost layer of the discourse dimensions (see “text” in Fig. 1 and the dark-grey field in Figs. 2 and 3) and two of the corners of the triangle, namely, those concerning the content and structures of the text.

Figure 3 is thus an attempt to present the theory of text movability. The different types of text movability mean that the reader has different focuses on the text, corresponding to different layers and corners in Fig. 3.

Sometimes Ronja comments spontaneously on what she is reading about in the book and relates it to her own values, experiences, and knowledge. “But you’re not allowed to do that?!” she exclaims in anger when she understands that Mimmi’s neighbour has tied the dog up outdoors at night. Ronja continues by wondering if she could ask her aunt, who is a vet, about the rules for keeping dogs tied. When Ronja reads on she gets the answer to her question, as Mimmi’s mother in the story phones a vet and finds out that a dog may only be tied up outdoors for 2 h. “There are people who get animals and then don’t bother about them just because they get tired and don’t think the animals are fun any more. If you have an animal you should look after it,” says Ronja. She thereby displays *associative text movability*, which means that the text and its content trigger the reader to go the innermost layer—the text—and relate it to her own experiences and ideas. What concerns the *content* of the book is related to the content of her own life—*what* the reader happens to think about during the reading, which can be related to the book. Associative text movability can also mean that the reader to some extent distances herself from the present text and says something about the *content* and *structures* of other texts through associations and connections to *what* other texts are about and *how* they are written. In associative text movability, then, the reader is geared to experiences outside the text, that is, in practices and texts in other practices (the middle and outer layers in Fig. 1, i.e., the two layers in Fig. 3) and the two corners in Fig. 3 which concern content and linguistic structures in other texts.

In this pilot study Ronja has not displayed the third and last category of text movability, *transactive text movability*. In this type of text movability the reader has moved still further from the text and talks about the context of the text, about things beyond the book and the envisionment building. This is seen when the reader thinks and talks about the purpose of the text and about other readers’ meaning-making in the text. This means that the reader can relate the content and linguistic structures of the text, for example, by talking about genre patterns and linguistic conventions to do with the context of the text. Moreover, the reader displays transactive text movability if he or she shows an awareness that features of language and content in the text may be more or less well suited to different readers. In transactive text movability the reader can move in all three layers in Fig. 1 and in all three corners in Fig. 3, which concern content, structures, and relations.

When Ronja read the three following books in the series about Mimmi and the pup, she was able to draw conclusions about structure and content, for example: “Bonita will handle this, because she usually does.” Such a statement can be interpreted as an incipient awareness of genres, saying that in books like this, individuals like Bonita will usually handle situations like this. Ronja also found the next three Mimmi books a little more difficult to read. When telling the dog handler: “But that’s good because you get better at reading as you go on”, she has definitely left the envisionment and gone beyond the specific text world.

The three figures and the discussion above concern the reader’s text movability and meaning-making in the envisionment building in the book. As we have already noted, Ronja also moves and makes meaning in the practice when she is *building envisionment with the book dog and its handler.* This envisionment, which Ronja builds together with the dog and the dog handler, proceeds from the *practice* they shape together.

## Ronja’s Practice Movability

When it comes to *the envisionment building in the practice with the book dog*, Ronja shows her engagement and her understanding when she puts down the book, scratches Arabella and brings the dog into the story by pointing and showing things to the dog, which she finds interesting in the illustrations. In the envisionment building in the practice with the book dog Ronja sees the dog as yet another reader in a sense, and imagines that the dog becomes engaged in the story. Ronja shows that she is convinced that Arabella can think and feel, and she talks to her and assumes that the dog is listening to the reading and relating to the text and her own life, in exactly the same was as Ronja herself does. Ronja is well aware that Arabella was found on the streets in Marbella in Spain and spent time in a dogs’ home while awaiting adoption. When the pup in the book is tied up outdoors, Ronja turns to Arabella, pats her and says in a comforting voice, “But you’re all right now.” The dog handler takes part in the envisionment building and adds first that “it was lucky that you were able to come to us and become a book dog instead of running around hungry in the streets” and then the dog handler also assumes the role of interpreter for the dog and says, in the dog’s voice, as it were: “I like being a book dog and listening when Ronja reads to me.” Ronja then turns to the dog and replies that she is a very good book dog and that it’s fun to read to her. Ronja’s practice movability thus shows that she is in the envisionment building with the dog and moves around there together with the dog handler, who supports Ronja’s envisionment building and text movability where the dog and the events in the book are involved.

The envisionment that Ronja builds with the dog and the dog handler proceeds from the *practice* they shape together. When Ronja shows her movability in practice with the book dog she does so by communicating with and establishing *relations* with the book dog and the dog handler about a *content* that is presented through *structures* in oral language, but also through, for example, gestures, glances, and other body language, with variations in speed and rhythm in speech and movements. The latter means that in the analysis of the practice movability we also apply multimodal perspectives and believe that even the dog in some sense can take part in the communication. The textual metafunction that concerns structures in language and text is thus expanded to comprise both verbal language and other meaning-making resources (Björkvall 2009). We want to stress here that when it comes to the envisionment building in the text it is the book that is in focus, while in the envisionment building in the practice with the book dog the focus is on the practice that the participants shape together, which in itself forms an expanded text.

**Fig. 4:**
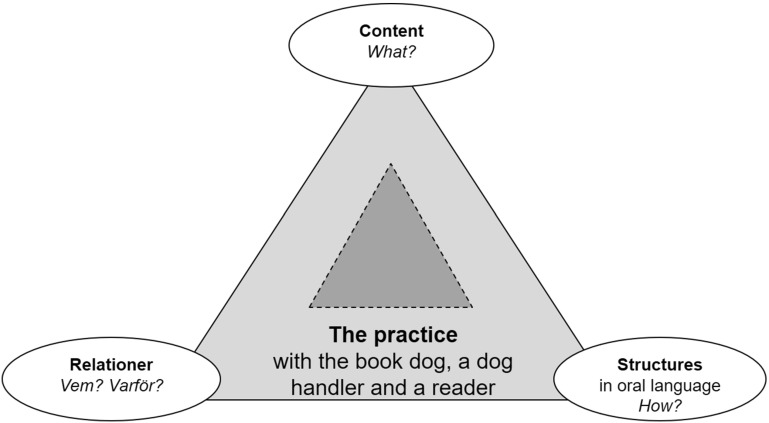
Movability in practice—envisionment building in the practice with the book dog (cf. Bergh Nestlog 2012, p. 68)

Figure 4 is an attempt to clarify the theories on which the analysis of the practice movability is based. In the figure the practice with the dog is represented by the light-grey field and the book (the text) by the dark-grey field. The book is thus part of the practice.

We call it *text*-*based practice movability* when the reader brings the book dog into the envisionment building and regards the dog as a reader, or rather as a listener with an interest in the content of the book. It has been found that Ronja tends to choose stories about dogs since she believes that this is something that interests the listening dog. Ronja says about Arabella, for example: “I know that she listens because when it gets exciting she licks my hand!” Arabella is happy and sometimes she leaves her sitting position for a while to lie on her back, rolling over and grunting with contentment. Ronja then draws the conclusion that the dog is reacting to something in the text and she acts as the dog’s interpreter: “Arabella thinks it’s getting scary,” she says, tickling the dog encouragingly on the stomach. The text-based practice movability can be compared with the text-based text movability, which is based on the reader talking about the text itself and thereby showing something of his or her understanding. It can happen that the text-based practice movability coincides with the associative text movability, as for example when the book is about a dog and the reader makes comparisons with the situation of the book dog and talks with the dog handler about that,^3^ and simultaneously brings the book dog into the envisionment building in the book; she can tell the dog not to be afraid of what is happening in the book, because “you don’t need to sleep outside any more, Bella.”^4^

Ronja loves animals and has well-formulated opinions about the value of animals and how they should be treated. She therefore treats Arabella with respect, talks to her and assumes that the dog is participating by listening to the reading. Arabella has received presents, a nice little glass jar that Ronja painted for her and an expressive drawing with glitter showing a girl with a dog. In this type of practice movability, the emotional relationship to the dog is important, not just in connection with the story in the book but also in life outside the envisionment building in the book. This illustrates what we call *associative practice movability*, in other words, when the reader links what is happening in the envisionment building in the practice with the book dog to her own knowledge, experience, and life outside the envisionment that is created through the reading.

Ronja also shows that she is able to distance herself from the envisionment she builds in the text and the practice with the dog. She does this when she talks about the importance of reading to the dog. She says that “[I]t’s the feeling! It’s so lovely to read to Bella. When I read to my teacher in school I just wonder the whole time if I’m doing right.” Here Ronja talks about two different discursive practices of which she has experience. One way to describe this distancing is to say that Ronja displays movability in practice, when she actually places herself outside the envisionment building of the text and the fantastic experience that she—and the dog—can enjoy there. Instead she talks about the purpose of reading with the dog, namely, that it is pleasant and not associated with demands as in school. We call this *transactive practice movability*, which can be compared with the transactive text movability that concerns, for instance, the reader’s understanding of the purpose of the text. Here the child shows the significance of the dog and the dog handler as a kind of structure for how the actual reading takes place.

In the study of the reader’s meaning-making together with the book dog we have related the three categories of text movability (text-based, associative, and transactive) to the metafunctions of social semiotic linguistics and the discourse dimensions in Figs. 1 and 2. Through Figs. 3 and 4 we have tried to show the connections between the different theories and deepen the discussion of the reader’s movability in the expanded envisionment building.

## Conclusions

In this study the aim has been to find theoretical perspectives which can explain Ronja’s reading together with the book dog, and to understand the significance of the book dog for the child’s reading. The study shows that Ronja movability is stretched out to an expanded envisionment building where both the book and the book dog are included.

One way to understand Ronja’s text movability and practice movability is that the book dog contributes with semiotic resources in Ronja’s meaning-making when she builds her conception of the content of the book. Through the dog she channels part of her understanding of the text. She creates a parallel envisionment building with the book dog in the discursive practice, the practice where she reads the book, and the dog contributes in its way with utterances to do with the reading. The dog can be seen as a medium between the text world and the child’s world, where the text world comes to life through the expanded envisionment building where the dog is central and highly palpable in reality. Ronja finds the reading with the dog a very positive experience, and by building an expanded envisionment through the text and the practice with the dog, her reading comprehension appears to be improved and her perception of herself as a reader is strengthened.

During the reading together with the book dog, Ronja has taken part in a social practice where the four resources that Luke and Freebody (1999) describe as essential for effective literacy development have interacted. Ronja has cracked codes, both large and small, when she has decoded the words and sentences of the text, and when she has experienced through the reading how a story can be structured and how she can link it to her own world view. By engaging in the expanded envisionment building she has created meaning in the text in a functional way, and at times she has critically analysed and questioned values in the book and the outside society.

We have not studied Ronja’s ability to decode, but the observations show that she gradually becomes more fluent in her reading. In the study we have not confined the analyses to matters concerning Ronja’s reading comprehension, but have extended the perspective to include the reading practice as a central and significant part of her reading and her meaning-making.

In the reading practice she has occasion to express herself orally and process her thoughts during the reading. The constantly listening dog is a tireless partner in conversation for the reader, who has infinite opportunity to formulate her understanding. Through the expanded envisionment building—the text and the practice with the dog—she also has expanded opportunities to make the envisionment real because the dog and the dog handler are there in reality.

This also means that she has greater potential to make linkages between envisionment and reality. Because she builds up the two envisionments in parallel, it is also reasonable to imagine that she has more thoughts than if she had only built envisionment in the book. Ronja demonstrates in different ways that she views reading as a meaningful activity in a meaningful and engaging context. There the book, the book dog, and the dog handler play important roles for her reading, and they support her in integrating the two envisionments with her own life as a person and a reader. When Ronja reads together with the book dog and moves in the two envisionments, it encourages her meaning-making in the text. The book dog functions as a support in getting involved in the book and giving her opportunities to talk about her meaning-making and thereby show some of her reading comprehension. In the envisionment building they share a literary experience where Ronja is needed to convey the text to the book dog.

The dog is, together with the book and the dog handler, a participant in that multimodal expanded text. In this multimodal text semiotic resources are used in the meaning-making during the reading process, and the resources has to be interpreted by the reader. For instance, when the dog pushes her nose towards the book or make a moaning sound, Ronja can see the dogs’ contribution as a sign for his deep interest in the story. Ronja also interprets the semiotic resources in the book, namely the written words and pictures. Furthermore, she interprets the dog handlers’ use of spoken words in different tones and volume, together with facial expressions. In the separate communication with the dog, the dog handler also uses sign language, certain words and tones, semiotic resources that the dog, but not the child, interprets. The dogs’ interpretation makes her respond in a way that is interpreted by the child. The three participants in the interaction use the semiotic resources each of them has access to, and their practice movability has different orientations: Ronja and the dog handler has focus on envisionment building in the text and in the practice. The dog handler is also orientated towards Ronja’s reading, the dog and the hidden communication with the dog. The dog has focus on the dog handler and the duty to show interest in Ronja and her reading. Their practice movability is characterised by the common interest in the relations to the others. In the interaction between the Ronja, the book dog, the dog handler and the book, meaning is created by the multimodal extended text that they build together with different semiotic resources in the discursive practice. In this way Ronja and the dog handler—and even the dog in some sense—show practice movability. Their assembled practice movability seem to support Ronja in her entire envisionment building in the text and in the extended text.

A central conclusion from the study is that the interaction in the discursive practice is empowering Ronja. She is the reader and the dog is the listener, and sometimes she has to explain what is said in the text and give her interpretation to the dog. The dog handler is all the time supporting the empowering potential by treating Ronja as a comprehensive and high qualitative reader and by using the common agreement in the silent contract in the interaction, the contract saying that the dog listens, speaks and understands. An example of this is the way the dog handler is asking Ronja questions either by using the dogs’ voice or by asking her to elaborate her interpretation of the text to the dog, as the dog handler has assumed that her elaboration is needed for the dog to fully understand. The power relationship that often exists between a reading child and a teacher or a parent is shifted and leveled out when the child may be the one who “teaches” and explains. To ask interview or control questions to the child in the reading situation would destroy the contract concluded. Instead the interaction following by the contract supports Ronja turning into a qualitative reader more or less comparable to the dog handler—or a teacher or a parent.

Through the study we have acquired a more profound knowledge of the significance of the reading practice for a child’s reading together with a book dog, which we regard as a medium that contributes with semiotic resources in the teaching situation. In future studies the children’s reading with book dogs will be examined through observations of reading practice and interviews with the children and dog handlers, and through analysis of texts which the children have written about their reading and about the books they have read.

The study also raises questions about other didactic matters. Pupils read texts in all school subjects and teachers shape their reading practices more or less consciously. What knowledge from the study with the book dog can be useful in other reading practices? Can the teaching offer possibilities for pupils to move in an expanded envisionment building where both text and practice stimulate their desire to invest in reading and expand and deepen their reading comprehension? As for the book dog’s engagement, attentiveness, and attitudes in connection with reading, there is a great deal to learn and think about concerning the reader’s potential to move in texts and reading practices.

## Footnotes

1 The project ran 2013–2016 in cooperation with Dyslexiförbundet FMLS i Nordvästskåne, and schools and libraries. The project focused primarily on children aged 6–13. The project leader was Author 2, who has continued the activity and research concerning book dogs since the project ended.

2 Both the “little code”, which has to do with letters, spelling, and other conventions, i.e., the usual structures of the written language, and the “large code”, which has to do, above all, with understanding how texts are used, why people write, the conventions for how different types of texts are written, and how contexts affect the way texts are used.

3 There are indications of associative text movability when the envisionment building in the book arouses associations with the reader’s own experiences.

4 There are indications of text-based practice movability when the reader imagines that the book dog understands what is happening in the envisionment building in the book.
